# DUR3 as a Molecular Lever for Coordinated Nitrogen and Phosphorus Uptake in Microalgae

**DOI:** 10.3390/biology15060452

**Published:** 2026-03-10

**Authors:** Geliang Ji, Xinyu Rui, Menghan Zhu, Yuqing Ma, Qing Shi, Enguang Nie, Long Wang, Haidong Ding, Jiahong Yu

**Affiliations:** College of Bioscience and Biotechnology, Yangzhou University, Yangzhou 225009, China; jgl20050824@163.com (G.J.); xinyur1204@gmail.com (X.R.); 18136929266@163.com (M.Z.); m13812063941@163.com (Y.M.); 18762320337@163.com (Q.S.); egnie@yzu.edu.cn (E.N.); wanglong@yzu.edu.cn (L.W.)

**Keywords:** *Chlamydomonas reinhardtii*, nitrogen–phosphorus co-uptake, transcriptomic analysis, urea transporter DUR3

## Abstract

Nitrogen (N) and phosphorus (P) are essential macronutrients for plant growth and major pollutants driving aquatic eutrophication. Microalgae represent a sustainable biological platform for nutrient recovery and circular utilization from wastewater; however, the molecular mechanisms governing efficient urea assimilation and its coordination with phosphorus uptake remain inadequately characterized. This study investigated how overexpression of the high-affinity urea transporter gene *DUR3* enhances nutrient scavenging capacity in the model green alga *Chlamydomonas reinhardtii*. The *DUR3*-overexpressing line exhibited concentration-dependent growth responses to urea, showing significant promotion at low-to-moderate levels but inhibition at high urea concentration or under pure-urea conditions, where *DUR3*-overexpressing (*DUR3*-OE) was more severely inhibited than the wild-type (WT). Notably, the *DUR3*-OE consistently increased chlorophyll content and photosynthetic efficiency (Fv/Fm) under ammonium, urea, and mixed-N regimes. Under low-urea conditions, the total P content of the *DUR3*-OE was 8.8% higher and total N content was 4.3% higher than in WT (*p* < 0.05). Except in pure-urea medium, the engineered strains exhibited significantly increased total P accumulation and superior P recovery efficiency from the culture medium. Transcriptomic analysis revealed that *DUR3* overexpression reprograms a coordinated regulatory network associated with N/P metabolism, photosynthesis, and carbon transport pathways. RT-qPCR validation confirmed significant upregulation of *PMA2* (plasma membrane H^+^-ATPase), phosphate transporters (*PTB3*, *PTB7*), the inorganic carbon transporter *HLA3*, and photosynthesis-related genes, which was associated with improved nutrient assimilation and photosynthetic performance. These findings establish *DUR3* as a key genetic target for engineering microalgae with optimized N-P co-uptake capacity, providing a robust molecular framework for developing high-efficiency algal strains for wastewater bioremediation and nutrient circular economy applications.

## Simple Summary

This study explored how to improve the ability of microalgae to clean wastewater by efficiently absorbing nitrogen (N) and phosphorus (P), which are essential for plant growth but can also cause water pollution. By focusing on the green alga *Chlamydomonas reinhardtii*, they discovered that overexpression of a specific gene, *DUR3*, significantly improves urea uptake. This enhanced absorption leads to better growth and optimizes photosynthesis under various N conditions. Moreover, it increases the alga’s capacity to simultaneously take up both N and P from mixed nutrient sources, although this P enhancement is absent under pure-urea conditions. The research uncovered that *DUR3* overexpression triggers a coordinated network of processes within the algae, including nutrient metabolism, photosynthesis, and carbon transport, leading to significant improvements in nutrient uptake efficiency. These findings highlight *DUR3* as a key genetic target for developing microalgae strains capable of efficient wastewater bioremediation and contributing to nutrient recycling, offering sustainable strategies for managing water pollution and resource recovery.

## 1. Introduction

Nitrogen (N) and phosphorus (P) are fundamental macronutrients essential for global food security and ecosystem productivity. Within wastewater streams, these elements represent valuable resources whose recovery aligns with circular economy principles and sustainable resource management. As a geologically finite resource with no synthetic substitute, P holds particular significance for closed-loop recycling strategies [1,2]. N serves as a core component of proteins, nucleic acids, and photosynthetic pigments, directly influencing biomass yield and metabolic activity [3]. Microalgal-based bioremediation has emerged as an environmentally sustainable platform that exploits photosynthetic metabolism to capture, assimilate, and convert N and P into value-added biomass for biofertilizers, animal feed, or bioenergy precursors. Engineering microalgal strains with enhanced nutrient scavenging capacity is therefore critical for maximizing nutrient recovery efficiency and ensuring robust performance across diverse wastewater matrices.

Among N species in aquatic environments, urea constitutes a major fraction of dissolved organic N in municipal and agricultural effluents, frequently accounting for 50% of the organic N pool at concentrations of approximately 150 µM [4,5]. As a concentrated and energetically efficient N source, urea presents significant potential for nutrient recovery in algal cultivation systems. Efficient utilization requires specific plasma membrane transporters to mediate cellular uptake prior to intracellular hydrolysis by urease. While this two-step process is well established, the contribution of urea transporter activity to overall N recovery efficiency in microalgae remains underexplored, particularly under mixed-N conditions that represent real-world wastewater environments.

Functional characterization of urea transporters has advanced significantly in higher plants. AtDUR3 in *Arabidopsis thaliana* mediates high-affinity urea uptake under N-limited conditions and contributes to internal N remobilization [6], while OsDUR3 in rice functionally complements urea transport deficiency in heterologous expression systems [7]. Notably, nutrient transporters in plants and microalgae often exhibit cross-regulatory capacity beyond their specific substrate transport functions, and such cross-regulation is a common mechanism for coordinated nutrient uptake; for instance, *OsNRT2.3b* overexpression enhances both N and P use efficiency in rice [8], underscoring transporters as potential nodes in nutrient coordination networks. In *C. reinhardtii*, intracellular urea catabolism is mediated by the urea amidohydrolase system (DUR1, DUR2) [9]. However, functional validation of DUR3 homologs in this microalga remains limited. Although genomic analyses have identified *DUR3*-like sequences in *C. reinhardtii*, their physiological contribution to urea acquisition and their potential integration within broader nutrient regulatory circuits remain undefined.

Coordinated acquisition of N and P is essential for photosynthetic organisms to maintain nutritional homeostasis under fluctuating environmental conditions. Cross-kingdom studies reveal intricate molecular crosstalk between N and P signaling networks, wherein perturbation of one pathway frequently triggers adaptive remodeling of the other. In *C. reinhardtii*, P starvation responses are orchestrated by the MYB-family transcription factor PSR1 (Phosphate Starvation Response 1), which activates expression of phosphatases (e.g., alkaline phosphatase encoded by PHOX) and high-affinity phosphate transporters, particularly members of the PTB (Phosphate Transporter B) gene family encoding Pi/Na^+^ symporters [10,11,12]. Additionally, the tonoplast-localized Pi-efflux transporter CrPTC1 (an SPX-SLC domain-containing protein) and CrVTC4 (an SPX-VTC domain-containing protein) regulate vacuolar polyphosphate homeostasis and intracellular phosphate storage dynamics [13]. Despite these advances in characterizing P metabolism, whether enhancement of a specific N uptake system such as urea transport can positively influence P acquisition in microalga remains unexplored. Given the variable N forms and N:P ratios characteristic of wastewater streams, elucidating such cross-regulatory mechanisms is essential for developing robust algal strains capable of efficient and balanced nutrient removal. We therefore hypothesize that augmenting urea transport capacity will trigger coordinated metabolic adjustments that enhance P scavenging and photosynthetic efficiency, thereby optimizing integrated N-P recovery.

Accordingly, this study investigates the physiological and molecular functions of the urea transporter DUR3 in *C. reinhardtii*. We characterize growth dynamics, cellular N/P quotas, photosynthetic performance, and transcriptomic responses of *DUR3*-overexpressing lines across environmentally relevant N regimes. By elucidating *DUR3*-mediated coordination of N-P metabolism, this work identifies a strategic genetic target for engineering microalgae with enhanced capacity for simultaneous nutrient recovery, thereby advancing the application of algal biotechnology in sustainable wastewater valorization and circular resource management [14].

## 2. Materials and Methods

### 2.1. Algae Strains and Growth Conditions

*C. reinhardtii* strain CC-4533 was used as the WT strain. The *DUR3*-OE strain (CSI_FC2D06) was obtained from the *C. reinhardtii* Resource Center [15]. Both strains were cultivated in TAP medium under mixotrophic conditions at 25 ± 0.5 °C, with a continuous light photoperiod (24:0 h light:dark), at an intensity of 50 µmol photons m^−2^s^−1^, and constant shaking at 150 rpm [16]. It should be noted that continuous light is a non-standard condition that may influence N metabolism and photosynthetic parameters.

### 2.2. Overexpression Identification

A single colony was picked and inoculated into 40 μL of 10 mM EDTA solution, followed by incubation at 100 °C for 10 min to perform crude genomic DNA extraction. PCR amplification was then conducted using the extracted DNA as a template with *DUR3* gene-specific primers JGL-DUR3-JD-F/R (Table S1). The resulting PCR amplicons were analyzed by agarose gel electrophoresis for qualitative identification to verify whether the selected algal colonies exhibited target gene overexpression. The positive algal colonies confirmed by this procedure were subsequently streaked onto fresh TAP solid medium for long-term preservation [17].

### 2.3. RT-qPCR

Next, 5–8 mL of homogenized algal culture was collected, and the suspension was subjected to centrifugation, with the supernatant completely discarded. The algal pellet was immediately snap-frozen in liquid nitrogen for 10–15 min, then rapidly transferred to a −80 °C ultra-low temperature freezer for long-term storage. Prior to RNA extraction, the samples were briefly refrozen in liquid N for 2 min to preserve the RNA integrity. Total RNA was isolated using the RNA extraction kit (TIANGEN Biotech Co., Ltd., Beijing, China) strictly following the manufacturer’s protocol.

After verifying that the RNA met the quality criteria for both purity and integrity, first-strand cDNA was synthesized via the two-step reverse transcription method using Hifair^®^III 1st Strand cDNA Synthesis SuperMix for qPCR (gDNA digester plus) (YEASEN Biotech Co., Ltd., Shanghai, China), a ready-to-use premix reagent integrated with genomic DNA removal function. The resulting cDNA product was stored at −20 °C for subsequent RT-qPCR analysis.

The RT-qPCR reaction was prepared following the standard protocol for Hieff^®^ qPCR SYBR Green Master Mix (No Rox) (YEASEN Biotech Co., Ltd., Shanghai, China). The entire preparation procedure was conducted on ice to prevent thermal inactivation of the DNA polymerase. The *CBLP* gene was used as an internal control [18]. The prepared reaction mixture was aliquoted into sterile, freshly opened 8-tube strips. After loading, the strips were gently centrifuged to ensure homogeneous mixing and remove air bubbles, and then placed into a quantitative real-time PCR instrument for amplification.

### 2.4. Experimental Design and Growth Parameters

All algal strains intended for subsequent experiments were subjected to homogenization treatment. Specifically, the target algae were picked from the solid medium and inoculated into the liquid medium for cultivation. Upon reaching the logarithmic growth phase, the algal culture was transferred into a fresh liquid medium at an inoculum size of 1% and this procedure was repeated 2–3 times to ensure the consistent growth status [19].

The experimental cultures were grown in 125 mL Erlenmeyer flasks containing 50 mL of specific culture medium. The experiments were carried out in a growth room under the same conditions described above. The experiment design consisted of five independent growth curves, each performed in triplicate for both biological and technical replicates, over a 4-day period. Cellular growth was monitored every 12 h by measuring absorbance at 750 nm (OD_750_ nm) using a spectrophotometer [20].

In addition, *C. reinhardtii* cells cultured to the logarithmic phase were subjected to serial dilution, followed by inoculation and cultivation on solid media with different nitrogen concentrations. The effect of *DUR3* overexpression on the growth status of *C. reinhardtii* was evaluated by comparing the colony color intensity between the WT strain and the overexpression strain on solid media.

Five growth conditions were tested: (a) 0U: 100% NH_4_^+^ (7 mM NH_4_Cl); (b) 0.1U: 10% urea (6.3 mM NH_4_Cl and 0.35 mM urea); (c) 0.5U: 50% urea (3.5 mM NH_4_Cl and 1.75 mM urea); (d) 0.9U: 90% urea (0.7 mM NH_4_Cl and 3.15 mM urea); and (e) 1U: 100% urea (3.5 mM urea) [21]. The TAP medium was initially prepared without any N source (TAP-N), and NH_4_Cl and urea were added according to the treatment design. The final N concentration in all media was maintained at 7 mM. The NH_4_Cl and urea solutions were sterilized by filtration on 0.22 µm pore size membranes before being added to the sterile culture medium.

### 2.5. Measurement of Total P and Total N

For the determination of total phosphorus (TP), the algal samples were first digested with concentrated nitric acid at 55 °C for 4–5 h, followed by quantification using ammonium molybdate-malachite green spectrophotometric method [22]. Absorbance was measured at 595 nm using a microplate reader, and the TP content was calculated based on the dry weight (DW), DW was obtained by centrifugation and drying at 55 °C in an oven for over 18 h [23]. The same ammonium molybdate-malachite green method was also applied directly to quantify P content in the culture medium. Total nitrogen (TN) in *C. reinhardtii* biomass was determined using the classical Kjeldahl method, selected for its high accuracy in microalgal N quantification. Protein content was subsequently calculated using a microalgae-specific conversion factor of 6.5 [24].

### 2.6. Chlorophyll Concentration Analysis

Chlorophyll a (Chl a) and chlorophyll b (Chl b) in *C. reinhardtii* cells were extracted in the dark using methanol as the solvent. The absorbance was measured at 665 nm and 652 nm using a microplate spectrophotometer, and the chlorophyll content in the cells was calculated according to established equations [25].

Chl a and Chl b concentrations (μg/mL) were calculated using the following equations:

Chl a = 16.29 × A_665_ − 8.54 × A_652_,

Chl b = 30.66 × A_652_ − 13.58 × A_665_,

Total Chl = 22.12 × A_652_ + 2.71 × A_665_.

### 2.7. Quantum Yield Analysis

Quantum Yield (QY) is a measure of the Photosystem II (PSII) efficiency. QY is equivalent to Fv/Fm in dark-adapted samples. Higher value indicates more efficient light energy conversion by PSII and a more stable photosynthetic function. For measurement, 10 mL of fresh algal culture at the logarithmic growth phase was transferred to a 10 mL sterile centrifuge tube, and the OD680 was adjusted to approximately 0.1 to ensure cell homogeneity. Samples were then sealed with a black light-shielding cover or placed in a dark box 20–30 min to fully close PSII reaction centers and eliminate interference from photoinhibition and fluorescence quenching [26]. The dark-adapted samples were transferred into a dedicated 4 mL cuvette for the AquaPen AP 110-C chlorophyll fluorometer (Photon Systems Instruments, Drásov, Czech Republic) ensuring no air bubbles, and QY value were measured. Each measurement was performed in triplicate biological replicates to minimize experimental errors.

### 2.8. Transcriptome Analysis

Both the WT and the *DUR3*-OE strains were cultured in TAP medium and grown to the logarithmic growth phase. Three biological replicates were prepared for each strain. Samples were quickly frozen in liquid N and stored at −80 °C until further use.

Samples were sent to Majorbio Bio-Pharm Technology Co., Ltd. (Shanghai, China) for transcriptome sequencing. Following quality control of the raw data, the clean reads were aligned to the *C. reinhardtii* reference genome (v5.5) using HISAT2 software (Version 2.2.1), a graph-based aligner optimized for accurate spliced transcript mapping [27]. Gene expression levels were quantified using RSEM software (Version 1.3.3) [28], which enables reliable estimation of gene and isoform expression from RNA-seq data. Differentially expressed genes (DEGs) were screened using DESeq2 software (Version 1.56.1) with a criterion of *p* < 0.05, a standard threshold for controlling the false discovery rate in differential expression analysis [29].

Gene Ontology (GO) and Kyoto Encyclopedia of Genes and Genomes (KEGG) enrichment analyses were performed, with particular focus on pathways related to N metabolism and P metabolism. This approach allowed exploration of the regulatory effects of *DUR3* overexpression on the transcriptome of *C. reinhardtii*.

## 3. Results

### 3.1. DUR3 Overexpression Identification

**Figure 1:**
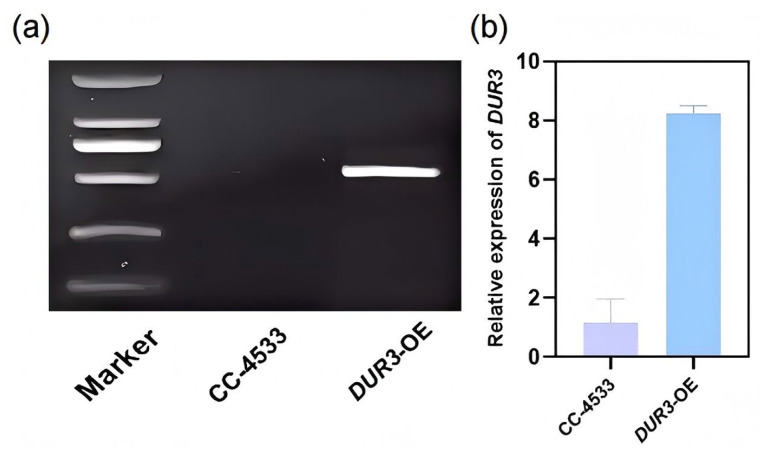
Verification of *DUR3*-overexpression strains. (**a**) Agarose gel electrophoresis electropherogram of post-PCR amplification products; (**b**) RT-qPCR analysis.

To validate the successful construction of the *DUR3*-OE, a two-step experimental approach was employed. First, PCR amplification was performed to confirm the genetic modification. Agarose gel electrophoresis of the PCR products revealed a distinct band of the expected size in the *DUR3*-OE, which was absent in the WT strain (Figure 1a and Figure S1). This result provided initial evidence that the *DUR3* gene had been successfully integrated into the genome of the *DUR3*-OE, confirming the construction of a stable *DUR3*-overexpression strain. To further verify the overexpression efficiency of *DUR3* gene, RT-qPCR was conducted using cDNA synthesized from total RNA as the template. The results demonstrated that the transcriptional level of *DUR3* in the *DUR3*-OE was 8-fold higher than in WT strain (Figure 1b). This finding directly confirmed that the *DUR3* gene was significantly overexpressed in the engineered strain, validating the effectiveness of our genetic modification strategy.

### 3.2. The Growth Between WT and DUR3-OE Strains

WT and *DUR3*-OE were used as research objects. Different urea concentration gradients were set up in a mixed nitrogen source system (urea + ammonium, final nitrogen concentration of 7 mM). Growth curves were plotted by measuring OD_750_ every 12 h to systematically investigate the regulatory effects of *DUR3* overexpression on nitrogen utilization efficiency and growth phenotypes of the strains.

**Figure 2:**
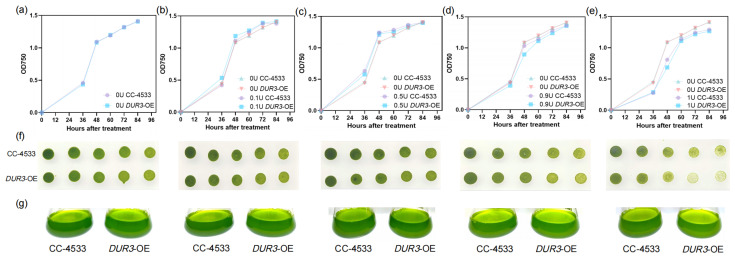
Growth phenotypes of the WT and the *DUR3*-OE under different urea concentrations in a mixed nitrogen source system. (**a**) Growth curve under 0U (no urea) treatment; (**b**) growth curve under 0.1U (low urea) treatment; (**c**) growth curve under 0.5U (medium urea) treatment; (**d**) growth curve under 0.9U (high urea) treatment; (**e**) growth curve under 1U (pure urea) treatment; (**f**) plate spotting assay; (**g**) phenotype of solution.

In the urea-free (0U) treatment (Figure 2a), the OD_750_ variation trends of the WT and the *DUR3*-OE were highly consistent, with no significant differences observed. Under low urea concentration (0.1U) treatment (Figure 2b), the growth advantage of *DUR3*-OE initially emerged: after 36 h, the OD_750_ growth rate of the *DUR3*-OE was significantly higher than that of WT at the same period. Compared with the inefficient absorption of low-concentration urea by WT, the *DUR3*-OE exhibited adaptive growth advantages under low urea ratio in the mixed nitrogen source. In the medium urea concentration (0.5U) treatment (Figure 2c), the growth difference between the two strains was most pronounced. During the logarithmic growth phase (36~48 h), the growth rate of the *DUR3*-OE was significantly faster than that of the WT. After 48 h, the OD_750_ value of the *DUR3*-OE reached 1.1~1.3, compared with ~1.0 in the urea-free treatment, corresponding to a biomass increase of up to 15.70%. Under high urea concentration (0.9U) treatment (Figure 2d), the growth of the *DUR3*-OE was significantly inhibited, whereas the growth inhibition of the WT was relatively mild. In the pure urea (1U) treatment (Figure 2e), the growth of both the WT and the *DUR3*-OE was significantly inhibited: after 48 h of cultivation, the OD_750_ value of the *DUR3*-OE was only 0.6~0.7, while the OD_750_ value of the *DUR3*-OE in the urea-free treatment at the same period was 1.0~1.1, corresponding to biomass reduction of over 30%.

The results of the plate spotting assay demonstrated that the growth performance of the WT and the *DUR3*-OE strains exhibited a distinct urea concentration-dependent pattern after inoculation on solid media supplemented with different nitrogen source ratios (Figure 2f). Specifically, both strains displayed the optimal growth status in the treatment group with a moderate urea concentration of 0.5U. In contrast, under high urea stress (0.9U and 1U), the growth performance of the *DUR3*-OE was significantly lower than that of the WT (Figure 2f). No significant difference in growth status was observed between the two strains under the N treatments (Figure 2f). These growth patterns were also visually apparent in the phenotypic performance of the two strains cultured in liquid medium (Figure 2g).

### 3.3. DUR3 Overexpression Improves Chlorophyll Content and Photosynthetic Efficiency

**Figure 3:**
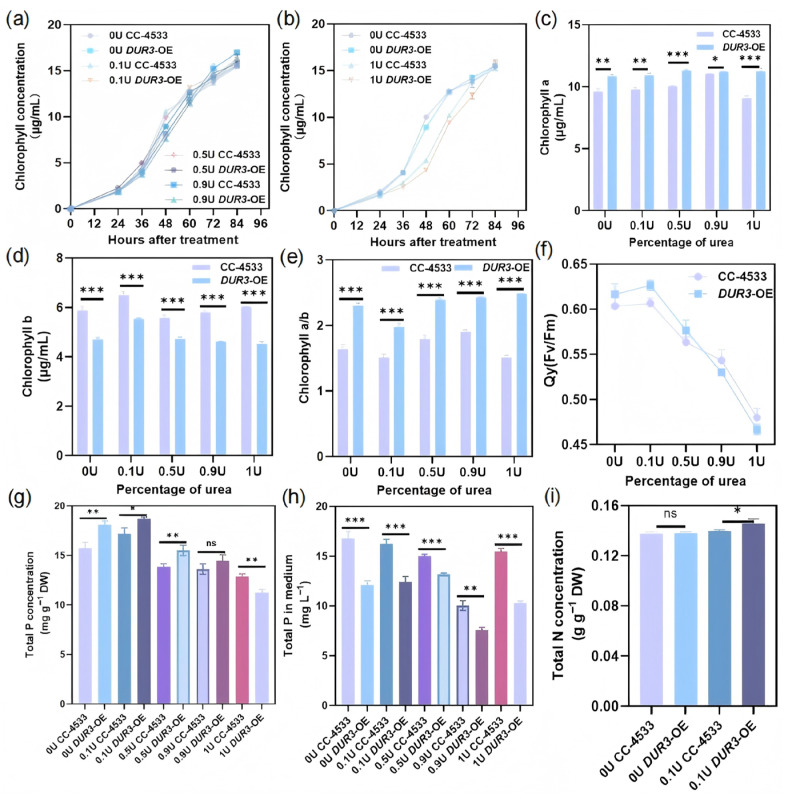
Biochemical analysis results of the WT and the *DUR3*-OE under different nitrogen source ratios. (**a**,**b**) Total chlorophyll content; (**c**) Chl a content; (**d**) Chl b content; (**e**) Chl a/b ratio; (**f**) Qy value (Fv/Fm); (**g**) total phosphorus content; (**h**) total P in medium; (**i**) total nitrogen content. Asterisks show a significant difference (Student’s *t*-test: * *p* < 0.05, ** *p* < 0.01 and *** *p* < 0.001). ns: not significant.

Except under the pure urea (1U) treatment where the total chlorophyll content decreased significantly, no significant differences in total chlorophyll was observed among the other N treatments (Figure 3a,b). This suggests that high concentrations of urea may inhibit chlorophyll synthesis or trigger stress responses in the photosynthetic system. At 84 h, the Chl a content of the WT was significantly lower than that of the *DUR3*-OE under all N source ratios, while the opposite was true for Chl b content, which was higher in the WT than in the *DUR3*-OE (Figure 3c,d). Meanwhile, we calculated the ratio of Chl a to Chl b and found that this ratio was significantly higher in the *DUR3*-OE than in the WT. A higher Chl a/b ratio indicates a structural adjustment of the photosynthetic apparatus in the *DUR3*-OE, specifically a relatively higher proportion of Chl a in the light-harvesting complex II of photosystem II, which is associated with enhanced light energy capture and conversion efficiency for photosynthesis (Figure 3e).

In the absence of urea (0U), the photosynthetic efficiency (Fv/Fm) of the two strains was comparable. When the urea concentration increased to 0.1U, the photosynthetic efficiency of the *DUR3*-OE strain was significantly upregulated, whereas WT showed only slight fluctuations. With further increase in urea concentration, the photosynthetic efficiency of both strains WT and *DUR3*-OE gradually declined (Figure 3f).

### 3.4. DUR3 Overexpression Improves Total N and P Content

The synergistic absorption of N and P nutrients is the basis for algae to maintain normal physiological metabolism. In this study, the total N and P contents of the *DUR3*-OE and the WT were measured under different N source ratios. The experimental results showed that in the medium only with ammonium chloride (0U), the *DUR3*-OE already exhibited a significant increase in total P content (Figure 3g), indicating that even when ammonium chloride was the sole N source, *DUR3* overexpression may promote P absorption and accumulation by activating the expression of P transporter genes. Upon addition of low-concentration urea (0.1U), total P content significantly increased in both WT and the *DUR3*-OE strains, with the *DUR3*-OE showing approximately 8.8% higher total P than the WT (Figure 3g). These results indicate that an appropriate ratio of mixed-N sources can synergistically activate the P uptake mechanism in algal cells, and enhanced urea absorption conferred by *DUR3* overexpression further strengthens this regulatory effect. With an increase in urea concentration, total P content of both strains gradually declined, but the *DUR3*-OE consistently maintained higher P levels than the WT, indicating a relative advantage in P uptake under medium to high urea concentrations. In contrast, under pure urea (1U) treatment, total P content of the *DUR3*-OE decreased significantly and was lower than that of the WT (Figure 3g). At 48 h of treatment, P content in the culture medium was also measured for all groups. The data demonstrated that the *DUR3*-OE line exhibited significantly lower P levels in the medium compared with the WT across all urea concentrations, suggesting that *DUR3* overexpression markedly enhances P uptake efficiency in algal cells (Figure 3h).

Total N content was determined in the 0U (only ammonium chloride) and 0.1U (low urea) treatment groups, where total P content was relatively high. The results showed that under pure ammonium chloride culture, there was no significant difference in total N content between the WT and the *DUR3*-OE (Figure 3i), indicating that when ammonium chloride was the sole N source, the N absorption advantage of *DUR3* overexpression could not be manifested due to the functional specificity of the DUR3 transporter. After adding of low-concentration urea (0.1U), the total N content increased in both strains, with the *DUR3*-OE showing approximately 4.3% higher total N than the WT (*p* < 0.05, Student’s *t*-test) (Figure 3i). Although this difference is modest, it is statistically significant and biologically meaningful: the slight but significant increase in cellular N synergizes with the enhanced P uptake in the *DUR3*-OE to optimizing the intracellular N/P stoichiometric balance, which is a key factor contributing to improved nutrient assimilation and growth performance of the *DUR3*-OE under mixed-N conditions.

### 3.5. Transcriptome Sequencing Analysis

To clarify the molecular mechanism by which *DUR3* overexpression regulates N and P accumulation in *C. reinhardtii*, RNA-seq was performed by Majorbio Bio-pharm Technology Co., Ltd. to compare transcriptomic differences between the WT and the *DUR3*-OE.

#### 3.5.1. Quality Control of Sequencing Data and Sequence Alignment Analysis

Raw sequencing data (38.82–47.25 million raw reads per sample) were filtered to remove adapters, low-quality reads, and sequences with high N content, yielding 38.81–47.24 million high-quality clean reads. Quality control showed that all samples had Q30 value ≥ 95.5% (base error rate ≤ 0.01%) and a GC content of 62–64%, indicating excellent data quality (Table S2). Clean reads were aligned to the *C. reinhardtii* reference genome (v5.5) using Hisat2, with ~97% of reads successfully mapped, including ≥93.5% uniquely mapped reads (Table S3), confirming the reliability of data for downstream transcriptomic analyses.

#### 3.5.2. Analysis of Gene Expression Levels and Differential Expression

**Figure 4:**
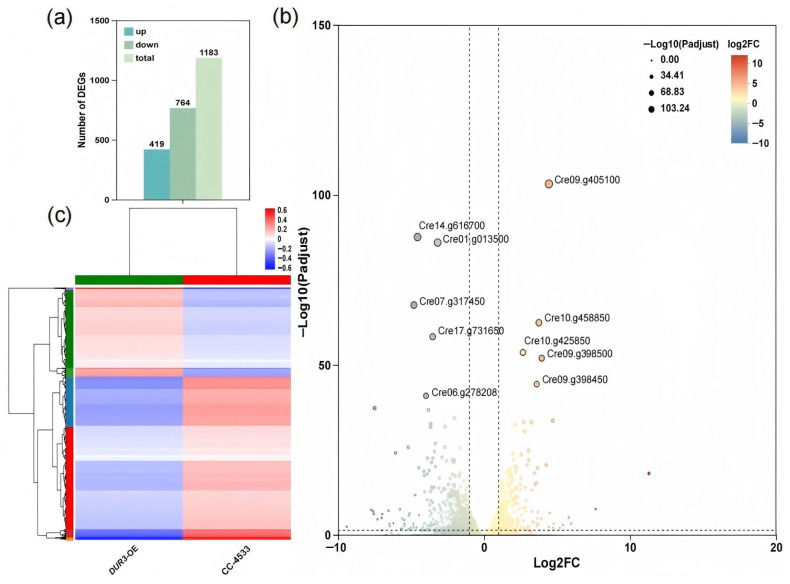
Transcriptome sequencing analysis results of the WT and the *DUR3*-OE. (**a**) The upregulation and downregulation of DEGs; (**b**) volcano plot of DEGs; (**c**) heatmap of differential expression genes.

Gene expression levels were quantified using RSEM with TPM (Transcripts Per Kilobase of exon model per Million mapped reads) as the standard index. A total of 14,945 validly expressed genes (TPM > 0) were detected, among which approximately 78% showing TPM values between 1 and 100, indicating a balanced and biologically reasonable expression distribution. Box plots could intuitively reflect the dispersion degree of gene expression levels within individual sequencing samples and simultaneously enable horizontal comparison of the overall gene expression levels among different samples (Figure S2). The correlation analysis of transcriptome samples between the WT and the *DUR3*-OE is presented in Figure S3, and the PCA analysis of the corresponding transcriptome data is displayed in Figure S4. Differentially expressed genes (DEGs) were screened using DESeq2 (*p* ≤ 0.05), resulting in 1183 DEGs including 419 upregulated and 764 downregulated genes (Figure 4a and Table S4).

Clustered heatmap analysis revealed distinct inter-group clustering of gene expression profiles between the WT and the *DUR3*-OE strains, with high intra-group consistency (Figure 4c). Volcano plots further identified core DEGs (e.g., Cre09.g405100, Cre14.g616700) with significant expression changes (Figure 4b). Notably, Cre13.g588150, encoding a vacuolar membrane phosphate transporter of the VTC family, was significantly upregulated in the *DUR3*-OE. As this transporter mediates the influx of cytoplasmic inorganic phosphate into vacuoles, its upregulation suggests a potential molecular mechanism contributing to the elevated total P accumulation observed in the *DUR3*-OE.

#### 3.5.3. EggNOG Annotation and Analysis of DEGs

EggNOG functional annotation showed that DEGs were predominantly enriched in functional categories related to N-P metabolism and cellular regulation, including signal transduction mechanisms, transcription, carbohydrate transport and metabolism, inorganic ion transport and metabolism, and amino acid transport and metabolism (Figure S5). These results suggest *DUR3* overexpression may enhance N-P uptake and transport efficiency by regulating key genes involved in these pathways.

#### 3.5.4. GO and KEGG Annotation Analysis of DEGs

**Figure 5:**
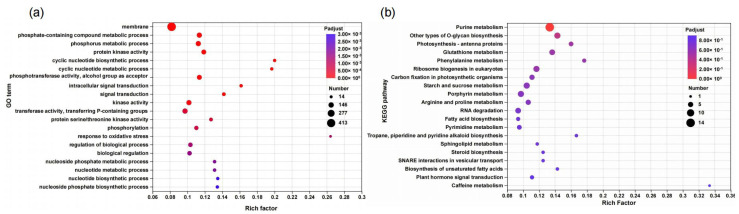
GO and KEGG enrichment bubble plots of the WT and the *DUR3*-OE. (**a**) GO enrichment bubble plots of DEGs; (**b**) KEGG enrichment bubble plots of DEGs.

GO functional enrichment analysis (Figure 5a and Figure S7) showed that DEGs were significantly enriched in terms closely associated with P metabolism and signal regulation.

In the cellular component (CC) category, DEGs were annotated to “cellular anatomical entity” and “protein-containing complex”, clarifying the spatial localization of functional genes involved in N-P metabolism. In the molecular function (MF) category, Enrichment was observed in “kinase activity”, “protein kinase activity”, and “transferase activity, transferring P-containing groups”, which are involved in phosphate group transfer and signal transduction, providing a molecular basis for P metabolic regulation. In the biological process (BP) category, core enriched terms included “phosphorus metabolic process” and “phosphate-containing compound metabolic process”, highlighting the involvement of DEGs in P uptake and metabolism. Additionally, terms such as “signal transduction”, “intracellular signal transduction”, and “phosphorylation” were enriched, indicating that *DUR3* overexpression may modulate N-P metabolism through signaling pathway activation.

To further reveal the molecular regulatory pathways mediated by DEGs and clarify their intrinsic association with the phenotypes of N and P accumulation as well as biomass increase induced by *DUR3* overexpression, KEGG pathway enrichment analysis was conducted on the screened DEGs. The KEGG results (Figure 5b and Figure S8) revealed several core pathways associated with the phenotypic changes observed in the *DUR3*-OE.

P metabolism-related pathways, “Purine metabolism” and “Starch and sucrose metabolism”, were enriched. Purine biosynthesis requires phosphorus-containing precursors (e.g., PRPP), and starch–sucrose metabolism involves phosphorylated carbohydrates, both of which promote P uptake and sequestration, consistent with the elevated total P content in the *DUR3*-OE strains. Photosynthesis-related pathway “Photosynthesis-antenna proteins” was significantly enriched, facilitating chlorophyll accumulation by enhancing light-harvesting efficiency, which aligns with the increased chlorophyll phenotype of the *DUR3*-OE strains. N metabolism-associated pathways, “Glutathione metabolism” and “Arginine and proline metabolism”, were enriched. These pathways utilize nitrogen-containing intermediates, and DUR3-mediated urea transport enhancement increases intracellular N supply, providing a material basis for the activation of these pathways. Meanwhile, nitrogenous metabolites generated in these pathways participate in N recycling and homeostasis, indirectly regulating global N metabolism. Carbon metabolism pathway “Carbon fixation in photosynthetic organisms” was enriched, promoting the synthesis of photosynthetic products and supporting biomass accumulation.

Of the 1183 DEGs, 764 were downregulated, accounting for 64.6% of the total. GO enrichment analysis revealed that these downregulated genes were significantly enriched in pathways related to P metabolism, kinase activity, and phosphorylation-mediated signal transduction (Figure S6). Which is highly relevant to the observed phenotype of increased P accumulation in this study. The widespread downregulation of kinase activity and phosphorylation-mediated signaling pathways may attenuate negative regulatory control of cellular P metabolism. Concurrently, the downregulated expression of P metabolism-related genes may reduce the consumptive utilization of P in secondary metabolic processes.

In summary, DEGs were predominantly enriched in pathways related to P metabolism, signal transduction, photosynthesis, and N metabolism. These findings suggest a coordinated regulatory network in which *DUR3* overexpression may link N metabolism adjustments to alterations in P and chlorophyll accumulation.

### 3.6. RT-qPCR Validation of Key Genes

**Figure 6:**
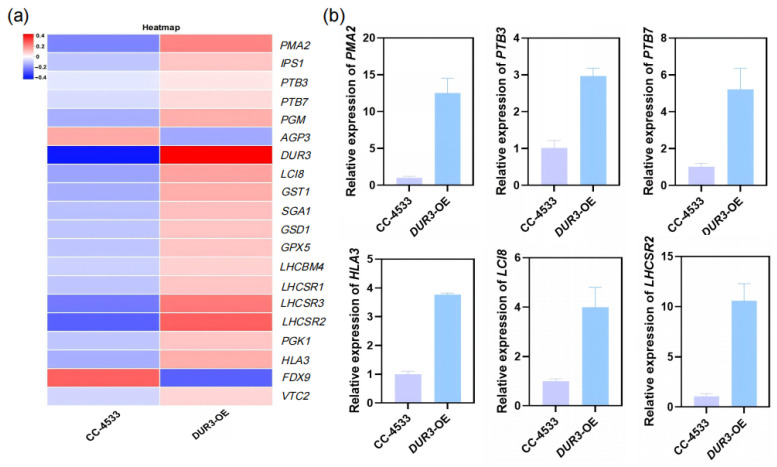
Heatmap analysis of key gene expression patterns and RT-qPCR validation. (**a**) Heatmap of key gene expression in the WT and the *DUR3*-OE; (**b**) RT-qPCR validation of some core genes.

To clarify the molecular mechanism by which *DUR3* overexpression regulates N and P metabolism in *C. reinhardtii*, we selected 20 key DEGs and separately constructed a heatmap for visual analysis (Figure 6a). These genes cover four functional categories closely related to the research focus: phosphate transport (*VTC2*, *PTB3*, *PTB7*), photosynthesis (*LHCSR1*, *LHCSR2*, *LHCSR3*, *LHCBM4*), nitrogen metabolism and stress response (*GST1*, *GPX5*), and carbon metabolism (*PGM*, *AGP3*, *SGA1*).

Heatmap results showed significant differences in expression of the selected key genes between the WT and the *DUR3*-OE. Among them, the *DUR3* gene was significantly upregulated in *DUR3*-OE, consistent with the earlier overexpression identification result (markedly increased transcriptional level). Phosphate transport-related genes *VTC2* and *PTB3* showed an upregulated trend; N metabolism and stress response-related gene *GST1*, as well as carbon metabolism gene *HLA3*, were also upregulated. Furthermore, photosynthesis-related genes *LHCSR1*, *LHCSR2*, *LHCSR3*, and *LHCBM4* were also upregulated in the *DUR3*-OE. The expression patterns of these genes are highly consistent with the phenotypes of enhanced N and P accumulation and optimized photosynthetic function in the *DUR3*-OE.

Furthermore, we selected partial key genes for RT-qPCR validation, and the results were highly consistent with the transcriptome sequencing data (Figure 6b). This validation result not only confirms the reliability of the transcriptome data but also provides direct experimental evidence for the molecular mechanism by which *DUR3* overexpression regulates N and P metabolism as well as photosynthetic function in *C. reinhardtii*.

## 4. Discussion

Microalgal-based nutrient recovery has emerged as a cornerstone strategy for advancing circular water economies by converting N and P pollutants in wastewater into valuable biomass for biofertilizers, feed, and bioenergy [30,31,32,33]. Despite promising pilot-scale demonstrations, the efficiency of algal remediation systems remains constrained by physiological imbalances particularly the asynchronous uptake of N and P which can lead to residual nutrient discharge or secondary pollution [34]. While engineering efforts have predominantly targeted single-nutrient pathways (e.g., overexpressing PSR1 for P removal) [35], strategies to achieve coordinated N-P scavenging through molecularly defined mechanisms remain limited. This gap is critical, as natural wastewater contains complex N speciation (urea, ammonium, nitrate), in which urea often dominates the organic fraction [36,37], yet its integration with P metabolism is poorly understood. Here, we identify *DUR3*, a high-affinity urea transporter in *C. reinhardtii*, as a previously unrecognized molecular lever associated with the coordinated regulation of N assimilation, P acquisition and carbon metabolism. Our work bridges a fundamental knowledge gap in algal nutrient signaling and provides a genetically precise solution for engineering strains with balanced nutrient recovery capacity.

The concentration-dependent growth phenotype of the *DUR3*-OE lines enhanced proliferation at low-to-moderate urea levels yet inhibition under high urea reflects the physiological trade-offs inherent in transporter engineering. At environmentally relevant urea levels, elevated *DUR3* expression accelerates urea influx, alleviating N limitation and supporting biomass accumulation, consistent with AtDUR3 function in *Arabidopsis* under N scarcity [6]. Conversely, growth inhibition under high urea aligns with observations in aquatic plants, where unregulated urea uptake overwhelms hydrolysis capacity, causing transient ammonium accumulation and metabolic stress [36]. This biphasic response underscores a critical design principle: transporter expression must be calibrated to target wastewater urea profiles to avoid metabolic imbalance a consideration increasingly emphasized in context-aware algal strain engineering [33,36,38].

Most significantly, *DUR3* overexpression consistently elevated cellular P content and P removal efficiency across multiple N regimes (excluding pure urea), revealing an unexpected regulatory nexus between urea transport and P metabolism (Figure 3). Transcriptomic and qPCR analyses confirmed coordinated upregulation of high-affinity phosphate transporters PTB3 and PTB7, canonical targets of the PSR1-mediated P starvation response [10,12]. This sustained *PTB* expression under non-starvation conditions contrasts sharply with the typical repression observed in wild-type *C. reinhardtii* [11], suggesting that enhanced urea-derived N flux generates a metabolic signal that modulates P sensing. Concurrent upregulation of *PMA2* (plasma membrane H^+^-ATPase) provides a compelling mechanistic link: by reinforcing the proton motive force, *PMA2* may energetically couple DUR3-mediated urea uptake (a proton-coupled process) with PTB-dependent phosphate symport, thereby establishing a synergistic ion-transport module (Figure 4, Figure 5 and Figure 6). To elaborate on this mechanistic link, although DUR3 is a dedicated urea transporter, its overexpression may induce a slight perturbation of the intracellular proton gradient, a common side effect of transmembrane protein overexpression in microalgae. This subtle proton gradient perturbation can activate the basal expression of *PMA2*, a key regulator of cellular proton motive force homeostasis; the upregulated *PMA2* then further transduces the signal to promote the transcriptional expression of downstream high-affinity phosphorus transporter genes (*PTB3*, *PTB7*), ultimately driving enhanced phosphorus uptake and intracellular accumulation in the *DUR3*-OE. This hypothesis aligns with prior evidence in microalgae, where *PMA* overexpression has been shown to enhance carbon fixation and lipid biosynthesis in *C. reinhardtii* [36], underscoring the central role of proton gradient regulation in coordinating nutrient uptake and carbon metabolism. Analogous cross-nutrient coordination occurs in higher plants; for instance, *OsA1* overexpression in rice improves both N and carbon use efficiency and enhances grain yield [39]. Nevertheless, our study represents the first demonstration in microalgae that engineering a single N transporter can systemically reprogram P acquisition networks, establishing DUR3 as a master regulatory node beyond its canonical transport function.

The concurrent enhancement of photosynthetic efficiency (Fv/Fm), chlorophyll content, and carbon-concentrating machinery (HLA3) underscores integrated metabolic coordination (Figure 3 and Figure 6). Elevated nitrogen availability fuels biosynthesis of photosynthetic apparatus components a well-documented nitrogen–photosynthesis relationship in microalgae [38], while *HLA3* upregulation ensures inorganic carbon supply meets enhanced photosynthetic demand [40]. This coordination aligns with cross-kingdom evidence of nutrient signaling crosstalk, yet our study uniquely demonstrates that engineering a single transporter (DUR3) can simultaneously optimize multiple metabolic modules. Critically, the absence of growth promotion under pure urea despite elevated nutrient quotas implies that balanced nutrient coordination rather than maximal accumulation, is physiologically decisive, reinforcing the importance of mimicking real wastewater conditions (mixed-N sources) in strain validation.

From an application standpoint, the *DUR3*-OE phenotype under mixed nitrogen conditions (0.1U) closely reflecting municipal wastewater composition [4] holds exceptional promise. Simultaneous elevation of cellular N and P content directly addresses the persistent challenge of stoichiometrically imbalanced nutrient removal in algal bioremediation. Unlike sequential treatment strategies requiring multiple engineered strains, DUR3-mediated coordination offers a genetically streamlined approach for synchronized N-P scavenging. Furthermore, enhanced P recovery efficiency positions the *DUR3*-OE as ideal candidates for producing P-rich algal biomass suitable for biofertilizer applications, a key objective in closing the P loop [1].

Although this study establishes *DUR3* overexpression as a promising strategy to enhance nitrogen–phosphorus co-accumulation in *C. reinhardtii*, several limitations warrant acknowledgment. First, the transcriptome sequencing analysis in this study was conducted under standard TAP medium log-phase conditions, rather than the mixed-N conditions where distinct phenotypic differences in algal growth and nutrient uptake were observed. While RT-qPCR validation confirmed that the expression trends of core N and P metabolism-related genes (e.g., *PTB3*, *PTB7*, *PMA2*) were highly consistent between standard TAP and mixed-N conditions, enabling indirect extrapolation of our transcriptomic conclusions to wastewater-relevant mixed-N regimes, direct transcriptome profiling under mixed-N conditions would yield more targeted molecular insights (Figure 6). Second, the mechanistic link between urea influx and *PTB/PMA2* induction remains correlative; future work employing *PSR1*-knockout backgrounds, phosphoproteomics, or promoter-reporter assays could elucidate the key signaling intermediates underlying this regulatory connection. Additionally, validation of the *DUR3*-OE strain’s nutrient uptake performance in real wastewater matrices containing organic inhibitors and variable pH is essential prior to any scale-up application. Furthermore, the long-term phenotypic stability of the *DUR3*-OE and its ecological fitness in polyculture systems both critical for practical bioremediation deployment also require comprehensive evaluation. Collectively, follow-up research integrating mixed-N condition transcriptome sequencing with multi-omics technologies, targeted genetic manipulation, and real wastewater validation will refine our understanding of *DUR3*-mediated N-P metabolic integration, and provide more robust foundation for engineering high-efficiency microalgal strains for sustainable wastewater bioremediation.

In summary, this study demonstrates that DUR3 is not merely a dedicated urea transporter, but also functions as a key regulatory node associated with the coordinated regulation of N and P metabolic processes in *C. reinhardtii*. By establishing DUR3 as a molecular lever for coordinated nutrient uptake, we provide both conceptual insight into algal nutrient signaling and a practical genetic target for engineering high-efficiency strains. These findings advance the paradigm of algal biotechnology from pollutant removal toward resource regeneration, offering a scalable molecular informed strategy to enhance circular nutrient recovery in wastewater valorization systems aligned with global sustainability goals.

## 5. Conclusions

(1) *DUR3* overexpression causes concentration-dependent growth responses to urea in *C. reinhardtii*;
(2) *DUR3*-OE enhances chlorophyll content, photosynthetic efficiency and N/P co-uptake under mixed nitrogen conditions;
(3) *DUR3* upregulation is associated with the expression of phosphorus transport and photosynthesis-related genes;
(4) DUR3 is a potential genetic target for engineering microalgae for wastewater bioremediation.

## Data Availability

The original contributions presented in this study are included in the article/Supplementary Materials. Further inquiries can be directed to the corresponding authors. The raw transcriptome sequencing data of the WT and the *DUR3*-OE in this study have been deposited in the NCBI database, with the BioProject accession number *PRJNA1419147*.

## Supplementary Materials

The following supporting information can be downloaded at https://www.mdpi.com/article/10.3390/biology15060452/s1, Figure S1: Original image of the agarose gel electrophoresis electropherogram of post-PCR amplification products; Figure S2: Box plot of transcriptome gene expression levels in WT and *DUR3*-OE; Figure S3: Correlation analysis results of transcriptome samples between WT and *DUR3*-OE; Figure S4: PCA analysis of transcriptome data from WT and *DUR3*-OE; Figure S5: EggNOG functional classification of DEGs between WT and *DUR3*-OE; Figure S6: GO enrichment bubble plots of downregulated DEGs; Figure S7: GO functional annotation analysis of DEGs between WT and *DUR3*-OE; Figure S8: KEGG pathway annotation analysis of DEGs between WT and *DUR3*-OE; Table S1: Primer sequences; Table S2: Quality control of sequencing data for WT and *DUR3*-OE; Table S3: Sequence alignment analysis results against the *C. reinhardtii* reference genome; Table S4: DEGs between WT and *DUR3*-OE.
