# Supplementary material for: DUR3 as a Molecular Lever for Coordinated Nitrogen and Phosphorus Uptake in Microalgae
Source: Biology (Basel). 2026 Mar 10;15(6):452. doi: 10.3390/biology15060452 (PMC13024400; doi:10.3390/biology15060452)
Supplement: Supplementary file 1 [file biology-15-00452-s001.zip › Figures S1-S8.pdf]

Figure S1-S8

S1

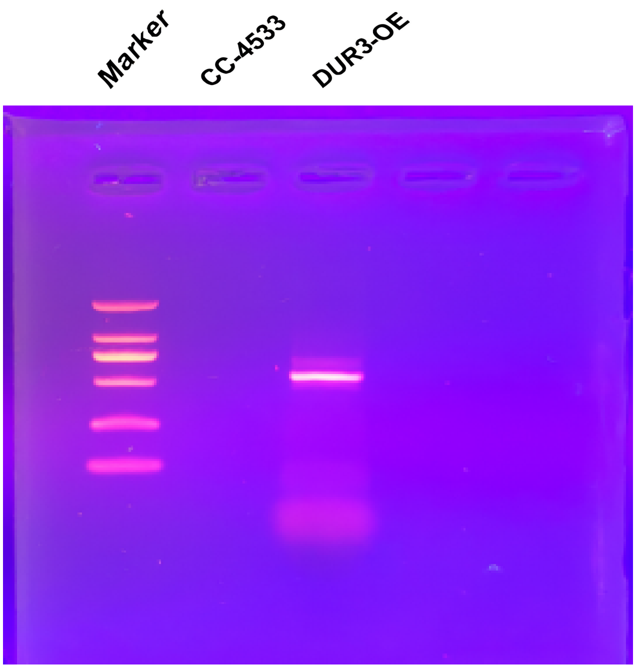

Figure S1. Original image of the agarose gel electrophoresis electropherogram of post-PCR amplification products.

S2

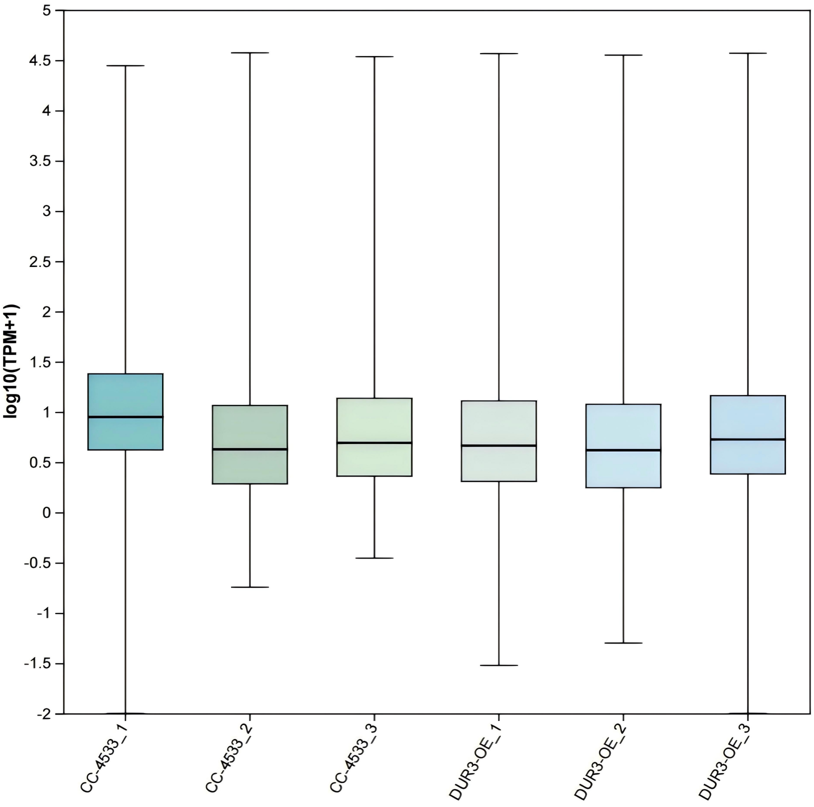

Figure S2. Boxplot of transcriptome gene expression levels in CC-4533 (WT) and *DUR3*-OE.

S3

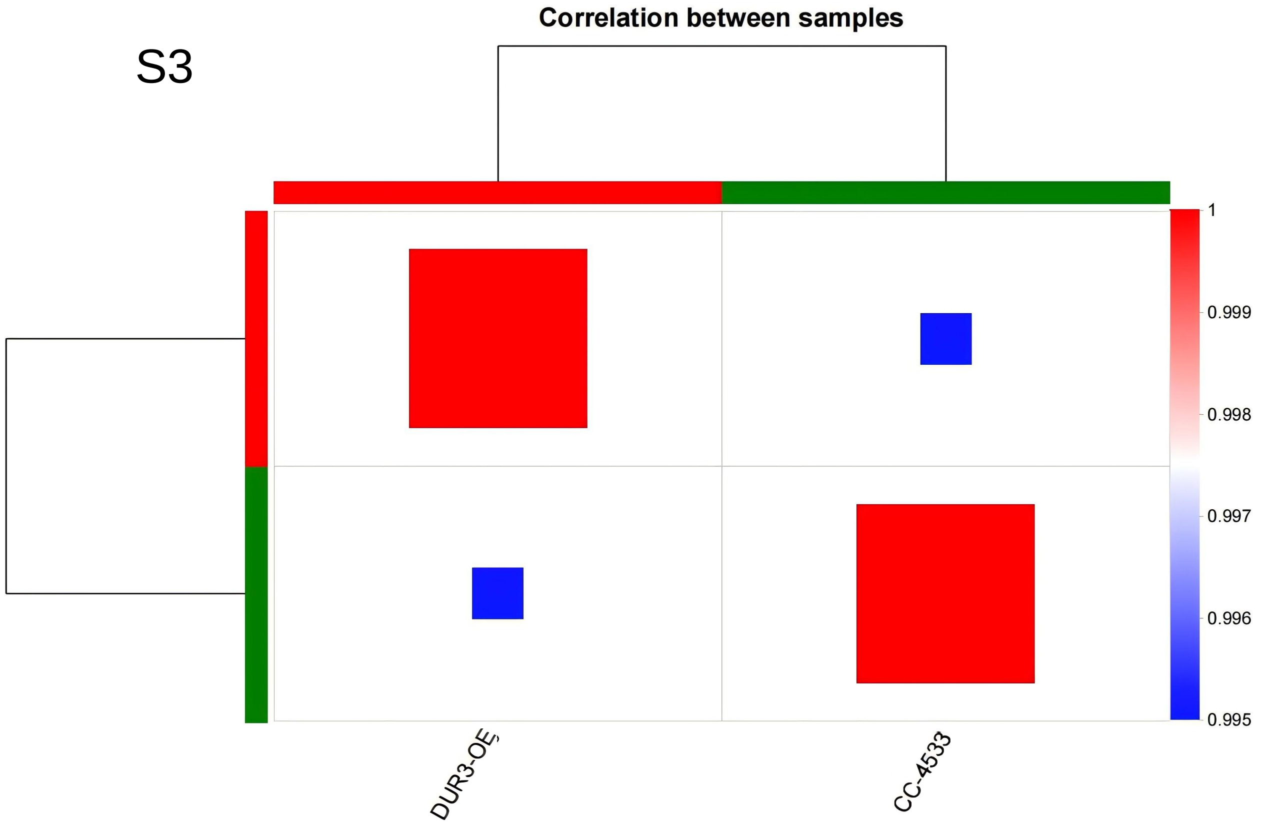

Figure S3. Correlation analysis results of transcriptome samples between CC-4533 (WT) and *DUR3*-OE.

S4

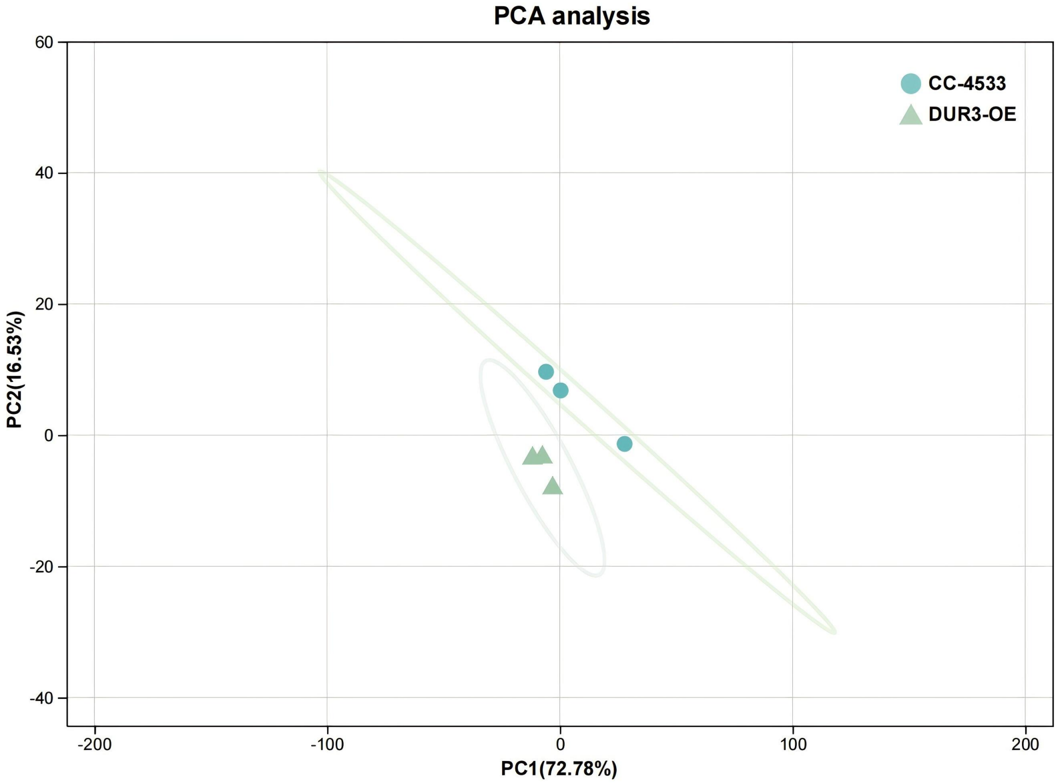

Figure S4. PCA analysis of transcriptome data from CC-4533 (WT) and *DUR3*-OE.

S5

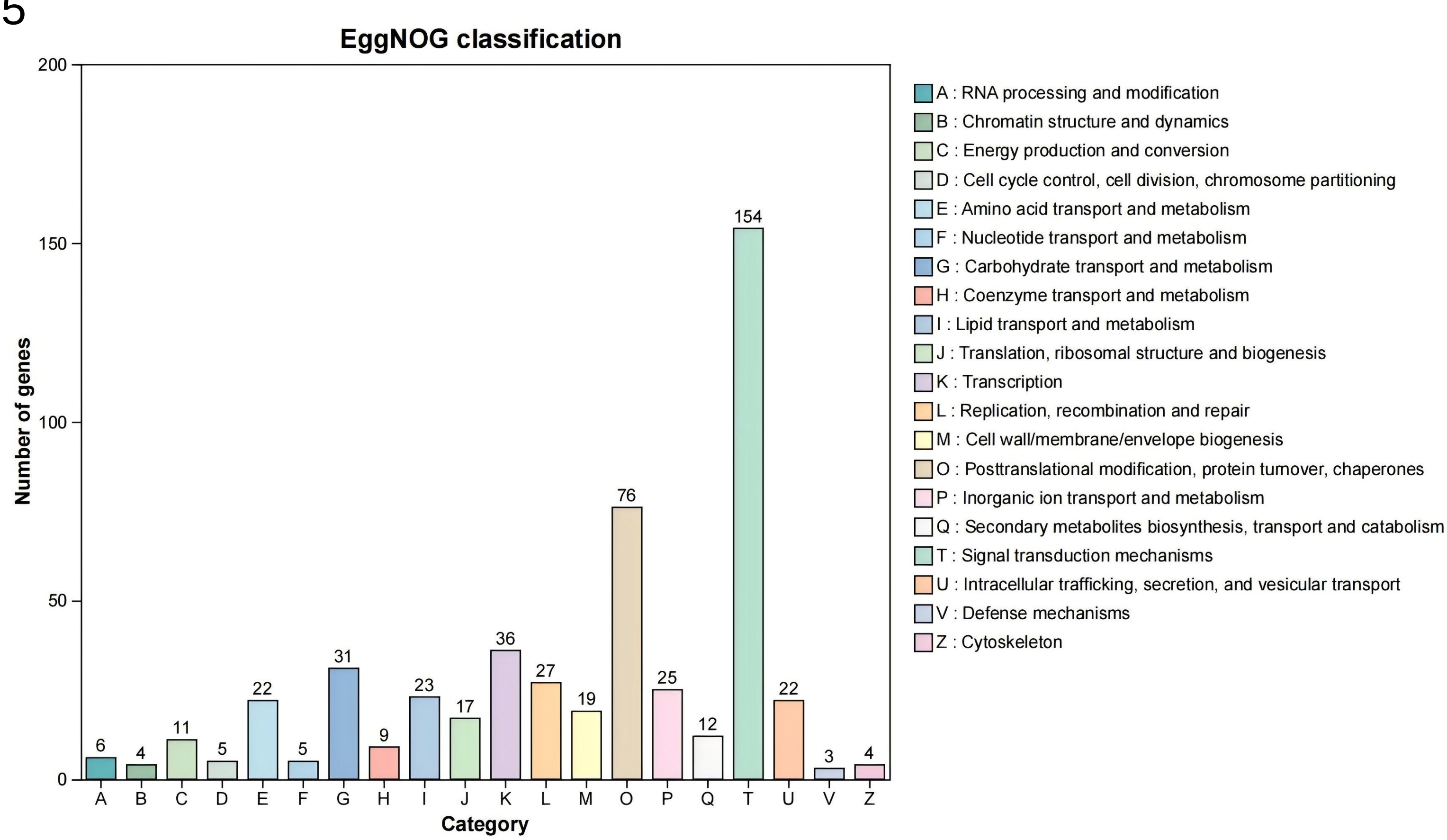

Figure S5. EggNOG functional classification of DEGs between CC-4533 (WT) and *DUR3*-OE. All genes shown in this figure are common to WT and *DUR3*-OE.

S6

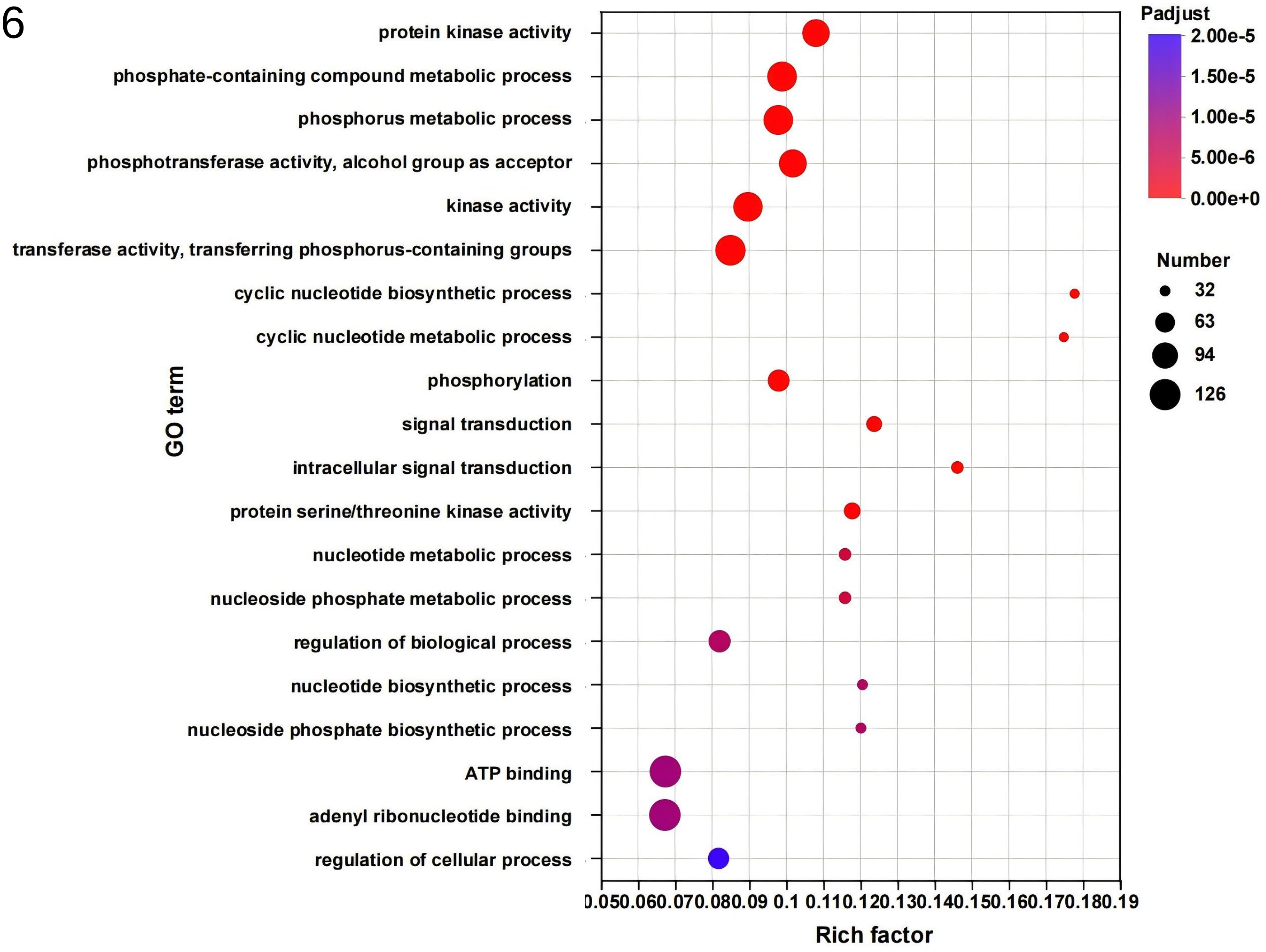

Figure S6. GO enrichment bubble plots of downregulated DEGs. All genes shown in this figure are common to WT and *DUR3*-OE.

S7

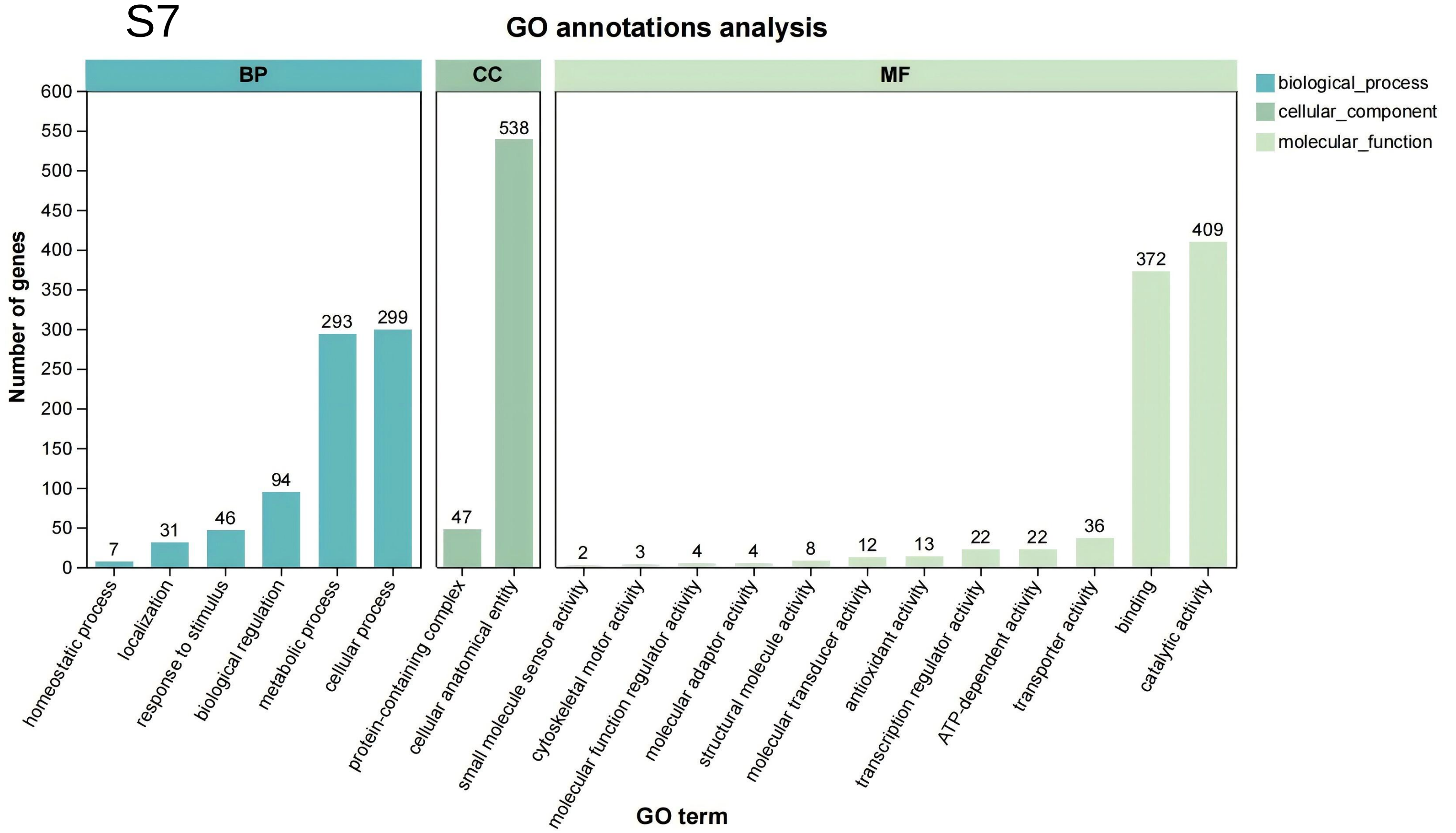

Figure S7. GO functional annotation analysis of DEGs between CC-4533 (WT) and *DUR3*-OE. All genes shown in this figure are common to WT and *DUR3*-OE.

S8

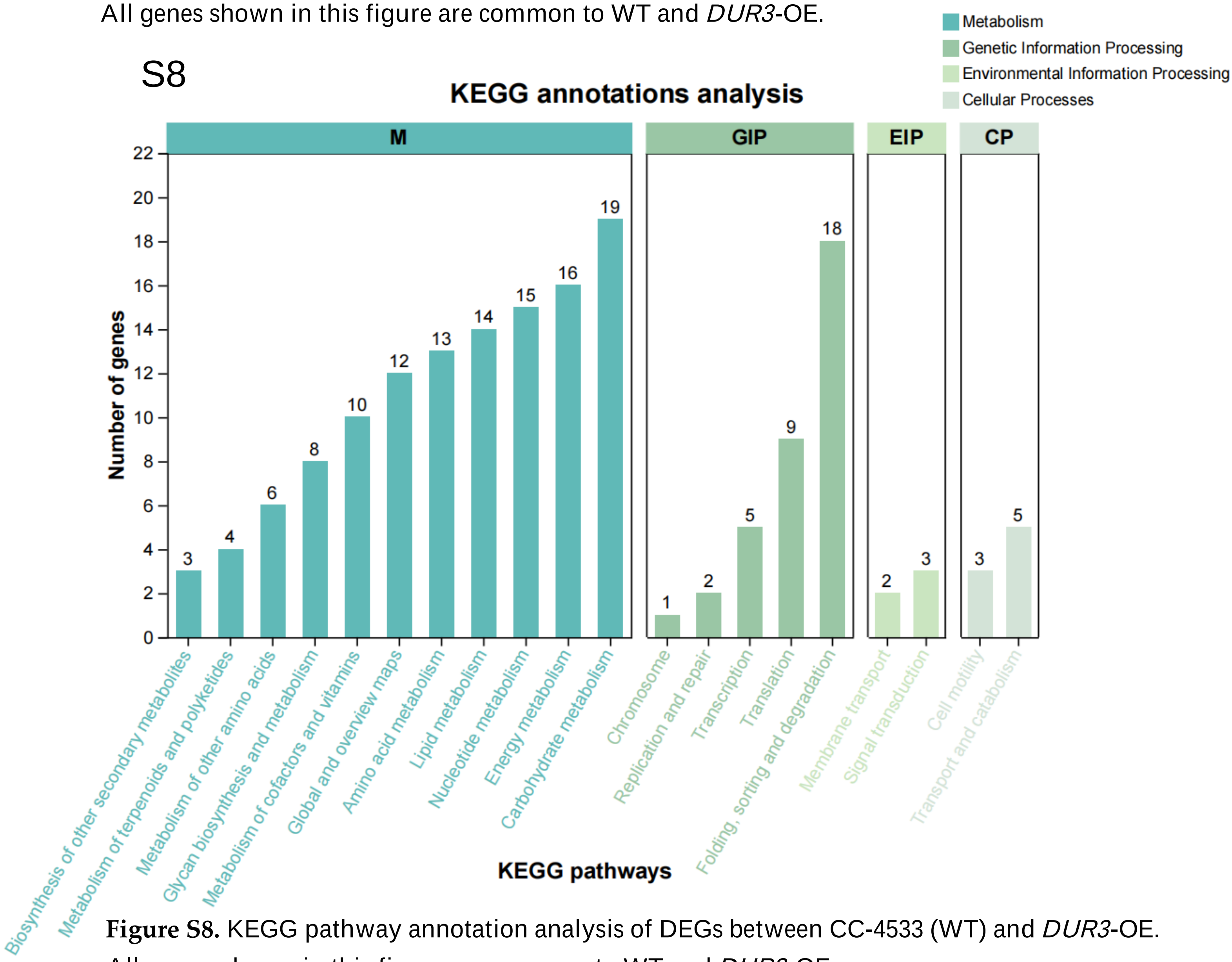

Figure S8. KEGG pathway annotation analysis of DEGs between CC-4533 (WT) and *DUR3*-OE. All genes shown in this figure are common to WT and *DUR3*-OE.
